# Mendelian Randomisation Analysis of Causal Association between Lifestyle, Health Factors, and Keratoconus

**DOI:** 10.3390/bioengineering11030221

**Published:** 2024-02-26

**Authors:** Jiaxuan Cheng, Lanting Yang, Yishan Ye, Lvfu He, Shihao Chen, Junjie Wang

**Affiliations:** 1 National Engineering Research Center of Ophthalmology and Optometry, Eye Hospital, Wenzhou Medical University, Wenzhou 325027, China; 2 National Clinical Research Center for Ocular Diseases, Eye Hospital, Wenzhou Medical University, Wenzhou 325027, China; 3 NMPA Key Laboratory for Clinical Research and Evaluation of Medical Devices and Drug for Ophthalmic Diseases, Eye Hospital, Wenzhou Medical University, Wenzhou 325027, China; 4 Department of Ophthalmology, Sichuan Mental Health Center, Mianyang 621054, China

**Keywords:** keratoconus, Mendelian randomisation, causal relationship, corneal biomechanics, Ocular Response Analyzer

## Abstract

Keratoconus (KC), a leading cause of vision impairment, has an unclear aetiology. This study used Mendelian randomization (MR) to explore the causal links between various factors (smoking, asthma, Down syndrome, inflammatory bowel disease, atopic dermatitis, and serum 25-hydroxyvitamin D levels) and KC. A two-sample MR design, grounded in genome-wide association study (GWAS) summary statistics, was adopted using data from FinnGen, UK Biobank, and other GWAS-related articles. The inverse-variance weighted (IVW) method was employed, complemented by the Wald ratio method for factors with only one single-nucleotide polymorphism (SNP). Sensitivity and stability were assessed through Cochrane’s Q test, the MR-Egger intercept test, MR-PRESSO outlier test, and the leave-one-out analysis. The IVW results for the ORA (Ocular Response Analyzer) biomechanical parameters indicated significant associations between tobacco smoking (CH: *p* < 0.001; CRF: *p* = 0.009) and inflammatory bowel disease (CH: *p* = 0.032; CRF: *p* = 0.001) and corneal biomechanics. The Wald ratio method showed tobacco smoking was associated with a lower risk of KC (*p* = 0.024). Conversely, asthma (*p* = 0.009), atopic dermatitis (*p* = 0.012), inflammatory bowel disease (*p* = 0.017), and serum 25-hydroxyvitamin D levels (*p* = 0.039) were associated with a higher risk of KC by IVW, and the same applied to Down syndrome (*p* = 0.004) using the Wald ratio. These results underscore the role of corneal biomechanics as potential mediators in KC risk, warranting further investigation using Corvis ST and Brillouin microscopy. The findings emphasise the importance of timely screening for specific populations in KC prevention and management.

## 1. Introduction

Keratoconus (KC) is a progressive, noninflammatory, and asymmetric disease, which leads to irregular astigmatism, corneal thinning, and eventually visual impairments. Early in the disease, patients are usually asymptomatic. However, as the disease progresses, visual acuity decreases and eventually the patient will notice distorted vision and a significant loss of vision [1]. In KC, the corneal stroma progressively thinning and losing structural integrity eventually leads to corneal bulging. Corneal stromal injury has been the main cause of corneal stromal thinning [2,3]. Meanwhile KC has become the most common cause of corneal transplants in both developed and developing countries [4,5] and its prevalence is reported to be between 0.2 and 4790 per 100,000 persons [6]. KC typically occurs and progresses rapidly in the second to fourth decade of life, but early symptoms of the disease can be subtle. However, if the disease is undiagnosed or untreated, the cornea may undergo local thinning and protrude into a conical shape. Therefore, the early diagnosis of KC and identification of its causal risk factors are critical for preventing the onset and progression of the disease.

The diagnosis of KC relies primarily on corneal tomography but now increasingly on corneal biomechanical parameters, given the fact that localised biomechanical decompensation often precedes diagnosable abnormality in corneal tomography [7]. Ocular Response Analyzer (ORA) and Corvis ST (CVS) are the only two clinical devices that facilitate in vivo biomechanical evaluations of the cornea and numerous studies have demonstrated enhanced diagnostic efficacy with biomechanical indices provided by these devices. The ORA applies an air puff to the anterior cornea, then records and measures the pressures at the two corneal applanation times [8]. CH (corneal hysteresis) and CRF (corneal resistance factor) are two important parameters measured by ORA. CH is an indicator of corneal viscous resistance, and CRF mainly reflects the combined effect of viscous and elastic resistance during corneal deformation. However, the causes of the biomechanical weakening in KC is undetermined, and in fact, the aetiology of KC, believed to be multifactorial with genetic and environmental factors, still remains elusive [1]. Observational epidemiological and cross-sectional studies have investigated the associations of smoking [9], asthma [1], Down syndrome [10,11], inflammatory bowel disease [12], atopic dermatitis [13,14], and serum 25-hydroxyvitamin D levels [15] with KC, but the findings bear uncertainties in the true causal relationship owing to limitations introduced by confounding factors and reverse causal associations.

The randomised control trial (RCT) is the gold standard in medical and scientific research. It is a research design that aims to evaluate the effects of a specific treatment, intervention, or strategy on a study group. RCT attempts to exclude confounding factors by randomly assigning participants to experimental and control groups, thereby producing evidence of a strong causal relationship [16]. But RCT experiments can be difficult due to time, cost, and ethical constraints. Meanwhile, Mendelian randomisation (MR) analysis is frequently considered analogous to a natural RCT [16]. It follows Mendel’s law of inheritance that “parental alleles are randomly assigned to the offspring” and genetic variation is unaffected by traditional confounding factors, such as environmental exposure, socioeconomic status, behavioural factors, or biomarkers [17]. Such characteristics mean that the effect of confounding factors and also the potential impact of reverse causation can be avoided with the MR analysis [18]. Therefore, the current study adopted a two-sample MR analysis approach to explore whether there was a causal association between aforementioned risk factors and KC. The study further included ORA parameters to investigate the roles of corneal biomechanics in KC development.

## 2. Methods

### 2.1. Study Design and Data Source

**Figure 1:**
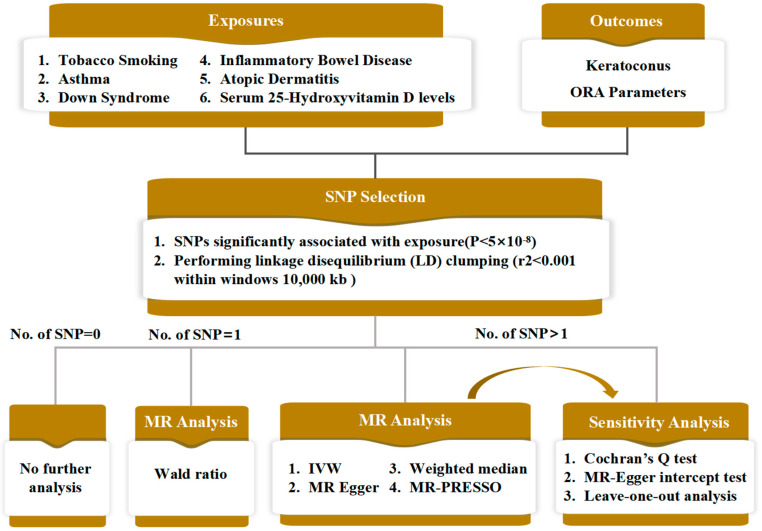
The schematic flow of the Mendelian randomisation analysis adopted in this study.

Due to the evidenced link between corneal biomechanical changes and KC development, the MR analysis of this study was divided into two parts: (1) explore the effects of exposure factors on corneal biomechanics by selecting ORA parameters (CH and CRF) as the outcomes; (2) explore the effects of these factors on KC directly by using KC itself as outcome. The exposure factors considered in this study included smoking, asthma, Down syndrome, inflammatory bowel disease, atopic dermatitis, and serum 25-hydroxyvitamin D levels (Figure 1).

All data used in the study were from the IEU Open GWAS project (https://gwas.mrcieu.ac.uk/, accessed on 3 July 2023). Genetic instruments obtained from the FinnGen database as exposure or outcome included keratoconus (311 cases and 209,287 controls; GWAS ID “finn-b-H7_CORNEALDEFORM”) and atopic dermatitis (7024 cases and 198,740 controls; GWAS ID” finn-b-L12_ATOPIC”). Summary statistics from UK Biobank were used as the data for current tobacco smoking, including 462,434 individuals of European (GWAS ID “ukb-b-223”) and 6572 individuals of African American or Afro-Caribbean (GWAS ID “ukb-e-1239_AFR”), ORA biomechanical parameters with corneal hysteresis (GWAS ID “ukb-b-11650”) and corneal resistance factors (GWAS ID “ukb-b-4717”) of the left eyes for 97,465 individuals of European ancestry, and asthma (GWAS ID “ukb-b-18113”) for 462,933 individuals of European ancestry. Genome-wide data for inflammatory bowel disease were from the International Inflammatory Bowel Disease Genetics Consortium (IIBDGC) published in 2012 (75,000 individuals of European ancestry; GWAS ID “ieu-a-292”) [19]. The data for the Down syndrome cell adhesion molecule were from a genome-wide association study of 3301 participants conducted by the Cardiovascular Epidemiology Unit (CEU) team at the University of Cambridge in 2018 (GWAS ID “prot-a-868”) [20]. Serum 25-hydroxyvitamin D levels were obtained from a UK Biobank genome-wide association study involving 417580 European individuals, and the median, mean, and interquartile ranges of serum 25-hydroxyvitamin D levels were 47.9, 49.6, and 33.5–63.2 nmol/L^−1^ (GWAS ID “ebi-a-GCST90000617”) [15].

Since all data involved in this study were obtained from the GWAS public database, no ethical approval or informed consent was required.

### 2.2. Data Analysis

An important step in the MR method is the determination of instrumental variables (IV) to carry out the analysis, and the validity of causal estimates with MR are dependent on three key assumptions: (1) the IV is unrelated to the typical confounding factors, (2) the IV is (reliably) associated with the exposure, and (3) the IV affects the outcome only through the risk factor [21]. The MR method thus uses genetic variants as IVs for assessing causal relationships from observational data [22]; meanwhile, single nucleotide polymorphisms (SNPs) are the most common genetic variant in the human genome. The development of high-throughput genomic technologies and genome-wide association studies (GWAS) has helped identify SNPs and determine genetic factors associated with complex diseases [23]. Therefore, SNPs that strongly related to each exposure factor were used as IVs in this study.

Figure 1 highlights the main steps for data analysis, which started with SNP selection, based on which various MR analyses was carried out, followed by sensitivity analyses. To ensure that the obtained SNPs were independent of exposure, linkage disequilibrium (LD) clumping (r^2^ < 0.001 within windows 10,000 kb for variants in the same locus) was used. SNPs were considered significantly related to the exposures if *p* < 5 × 10^−8^, and these SNPs were then adopted as IVs. To assess the association strength between the IV and the exposure factor, the F value was calculated for each IV in the form of $R^{2}\times \left(N-2\right)/(1-R^{2})$ when N, the sample size of the GWAS for each exposure, was available and $\beta_{\mathrm{exposure}}^{2}/{\mathrm{se}}_{\mathrm{exposure}}^{2}$ when N was not available; here, $R^{2}$ was estimated as $2\times \beta_{\mathrm{exposure}}^{2}\times {\mathrm{EAF}}_{\mathrm{exposure}}\times (1-{\mathrm{EAF}}_{\mathrm{exposure}})$, where ${\mathrm{EAF}}_{\mathrm{exposure}}$ was the effect allele frequency and $\beta_{\mathrm{exposure}}$ and ${\mathrm{se}}_{\mathrm{exposure}}$ were the estimated genetic effects on the exposure factor in question [24,25]. IVs with F values < 10 were excluded for further analysis.

MR analysis methods were applied as follows [26]: IVW, MR-Egger, weighted median, and MR-PRESSO were applied and scatter plots of the analysis results were also plotted for visualisation when the numbers of SNPs were >1. The Wald ratio was used if only a single SNP was identified, whereas no further analysis was carried out for exposure factors with no identified SNP.

Because genetic variants may affect the outcome through pathways other than through the risk factor of interest (so called horizontal pleiotropic effects). The intercept from the MR-Egger analysis can be interpreted as the average pleiotropic effect of a genetic variant included in the analysis [27,28]. *P* > 0.05 for the MR-Egger intercept indicates that no evidence of horizontal pleiotropy exists. Cochran’s Q statistic was used to assess heterogeneity of the IVs and *p* > 0.05 means that no significant heterogeneity exists for these associations. The MR-PRESSO analysis was used for outlier detection [29], and the MR analysis, heterogeneity testing, and horizontal testing were performed again after removing outliers if detected (Figure 1). The leave-one-out analysis was used to test the stability of the MR results. For IVs containing only a single SNP, the sensitivity analysis outlined herein could not be performed.

All analysis was conducted using the TwoSampleMR package (version 0.5.7) in R language (version 4.3.0). *p* < 0.05 was considered statistically significant.

## 3. Results

### 3.1. Mendelian Randomisation Analysis with ORA Parameters as the Outcomes

The results of the two-sample MR analysis examining the causal associations of tobacco smoking and inflammatory bowel disease with the ORA parameters are listed in Table 1 and Table 2. The analysis indicated no associations between other exposures and the ORA parameters (i.e., 0 SNP).

Outliers for inflammatory bowel disease (CH: rs6920220 and rs7240004; CRF: rs7240004 and rs12142199) were detected by MR-PRESSO; therefore, the results in the tables are based on analysis after removing the outliers.

MR estimates indicated that tobacco smoking was significantly associated with ORA parameters susceptibility using the IVW (CH: OR = 1.572, *p* < 0.001; CRF: OR = 1.380, *p* = 0.009). Such causal association was also presented in the scatter plot (Supplementary Figures S1a and S2a). The leave-one-out analysis suggested that the causal effect results of tobacco smoking and ORA parameters in the IVW analysis were not driven by any single SNP (Supplementary Figures S1b and S2b), indicating the stability of the analysis. Similarly, the IVW (CH: OR = 0.989, *p* = 0.032; CRF: OR = 0.982, *p* = 0.001) showed a causal relationship between inflammatory bowel disease and ORA parameters (Supplementary Figures S3a and S4a). Moreover, in the MR-Egger intercept test, no horizontal pleiotropy was found for SNPs used in tobacco smoking (CH: *P*-pleiotropy = 0.192; CRF: *P*-pleiotropy = 0.624) and inflammatory bowel disease (CH: *P*-pleiotropy = 0.722; CRF: *P*-pleiotropy = 0.879). Collectively, these findings suggest that tobacco smoking and inflammatory bowel disease affect corneal biomechanical properties.

### 3.2. Mendelian Randomisation Analysis with Keratoconus as the Outcome

Table 3 showed the results for the causal associations between KC and other risk factors, including current tobacco smoking, Down syndrome, asthma, atopic dermatitis, inflammatory bowel disease, and serum 25-hydroxyvitamin D levels. The Wald ratio (OR = 0.055, *p* = 0.024) method showed that tobacco smoking is associated with a lower risk of KC. Instead, asthma (OR = 39.901, *p* = 0.009), atopic dermatitis (OR = 1.452, *p* = 0.012), inflammatory bowel disease (OR = 1.206, *p* = 0.017), and serum 25-hydroxyvitamin D levels (OR = 2.146, *p* = 0.039) were associated with a higher risk of KC by the method of IVW. The Wald ratio (OR = 3.276, *p* = 0.004) showed statistical significance between Down syndrome and higher risk of KC. The MR-Egger intercept test did not yield any indication of directional pleiotropy for the association between these factors and KC (all *P*s > 0.05), and Cochran’s Q test revealed no significant heterogeneity exists for these associations (all *P*s > 0.05), indicating the reliability of the causal results. The causal associations were presented in the scatter plots, and the leave-one-out analysis suggested that the causal effect results of these risk factors and KC in the IVW analysis were not driven by any single SNP (Supplementary Figures S5–S8).

## 4. Discussion

This study examined causal associations between several potential influencing factors and KC using a broad set of variables obtained from the largest genetic databases currently available. Two sample MR analysis showed that there was a causal relationship between tobacco smoking and lower risk of KC, while Down syndrome, asthma, atopic dermatitis, inflammatory bowel disease, and Serum 25-Hydroxyvitamin D levels had causal associations with higher risk of KC. Causal associations of tobacco smoking and inflammatory bowel disease with the ORA parameters was also observed. Collectively, it can be speculated that changes in corneal biomechanical properties may be an intermediate factor that prevents or leads to the development of KC.

In recent years, MR analysis has been gradually gaining research attentions with its new perspective of inferring the causal relationship between exposure and outcome [16,18]. A number of MR studies have identified potential causal associations between lifestyle or biological exposures and ocular diseases, thus providing an opportunity for further pathological studies and interventional development [30,31,32]. To the best of the authors’ knowledge, this was the first study to estimate potential risk factors for KC by using the GWAS database.

The study indicates that there is potential genetic evidence for a causal relationship between tobacco smoking and the lower risk of KC, after excluding confounding factors and reverse causal associations, which are consistent with the observational studies. Hafezi et al. showed a statistically significant increase in CH and CRF in smokers, which suggested that chronic smoking might have a beneficial effect on corneal biomechanics [33], because conversely the reduction in corneal biomechanical properties were thought to play an important role in the progression of KC [34], and studies indicated a lower CH and CRF in KC [8,35]. A study by Sahebjada et al. suggested KC was associated with smoking using univariate regression analysis but that association became statistically insignificant following a multivariate regression analysis [9]. While these observational studies showed partially significant associations between smoking and KC, they had their own limitations related to potential uncontrolled confounders that might mask the true associations. The design of the current study could otherwise overcome these limitations.

Although KC is traditionally considered a non-inflammatory disease, some inflammatory molecules have been found in the tear fluid of patients with KC, suggesting that immune-related diseases factors may be associated with the pathogenesis of KC [36]. In a study among Dutch residents, KC appeared to be positively associated with a variety of immune-mediated diseases, including asthma and inflammatory skin conditions [37]. Sahebjada et al. have also highlighted a significant association between asthma and an increased risk of severe KC [9]. An observational study involving of 4272 adults with severe atopic dermatitis demonstrated a notably high hazard ratio (HR) of 10.01 (95% confidence interval [CI], 5.02–19.96) for developing KC [38]. In contrast, a recent MR analysis, based on a different database other than the one in the current study, did not identify a significant causal effect of atopic dermatitis on KC [39]. Nevertheless, the current study aligns with previous observational findings, indicating that asthma, inflammatory bowel disease, and atopic dermatitis were positively associated with KC risk.

The main clinical manifestation of KC is corneal thin and ectasia, phenomena largely ascribed to the degradation of the extracellular matrix (ECM) [40]. MMPs (matrix metalloproteinases), which are zinc-dependent endopeptidases secreted by epithelial cells, stromal cells, and neutrophils, play a critical role in this process by cleaving components of the ECM, such as collagen and elastin [41]. Current evidence indicates that KC patients exhibit elevated levels of inflammatory molecules, including interleukin-6 (IL-6) and tumour necrosis factor-α (TNF-α), in their tears [42,43]. Furthermore, several studies have established that IL and TNF regulate the expression of MMPs [14,40,42]. This suggests that chronic inflammatory processes are integral to the pathogenesis of KC.

Meanwhile, inflammatory bowel disease, including Crohn’s disease and ulcerative colitis, is a chronic inflammatory disorder of the intestines of unknown aetiology. In inflammatory bowel disease, tissue damage is primarily driven by adaptive immunity, and the adaptive response to specific antigens is influenced by a combination of resident and recruited cell populations. This includes the release of cytokines such as IL-1, IL-6, and TNF-α by macrophages [12,44]. Furthermore, Vitamin D plays a role in modulating both innate and adaptive immune systems, and its deficiency is associated with allergic disorders [45,46,47]. 

These findings collectively elucidate the causal relationship between KC and immune-related factors, including asthma, atopic dermatitis, inflammatory bowel disease, and serum 25-hydroxyvitamin D levels.

The study also indicated a causal association of Down syndrome with higher risk of KC. Concurrently, Maria et al. found that patients with Down syndrome had more than six times higher odds of KC than those without this condition [13]. A study, which reported the corneal morphologic characteristics in a large series of patients with Down syndrome, showed that patients with Down syndrome had steeper and thinner corneas and more corneal aberrations than those without genetic alterations and normal corneas [48]. Some studies have shown that Down syndrome was associated with more frequent eye rubbing, which may contribute to the high prevalence of KC among persons with Down syndrome [49,50].

Although MR has many advantages in causal inference and can provide a complement to traditional observational studies, several limitations of the current study need to be noted. Firstly, the study was limited to individuals of European ancestry and the conclusions may not be fully applicable to other populations. Secondly, the robustness of the results for two exposures (namely, smoking and Down syndrome) was constrained because these exposures only had a single SNP. Thirdly, the KC data included in this study did not distinguish between gender and age. However, the recent studies suggest that sex hormones can maintain corneal structural integrity by influencing wound healing and corneal stromal thickness, and that prolactin-induced protein is an important hormonally regulated biomarker in KC [51,52]. Similarly, KC usually progresses rapidly in youth. Therefore, the further division of KC population according to gender and age before MR analysis will yield more accurate results. Meanwhile, the purpose of this study was to investigate the risk factors for KC and to use this as a reference for clinical prevention and screening. Currently, the main treatments for KC are rigid gas permeable, corneal cross-linking, etc. [53]. These treatments can control the progression of KC, so early diagnosis of KC and effective treatment measures will reduce the risk of corneal transplantation in patients. Lastly, the mechanism behind the causal association between smoking and KC was not directly proven. Instead, the inference was made through the ORA parameters, the only in vivo corneal biomechanical parameters available in the database. However, they may not be the most accurate representation of corneal biomechanics, and in fact, the study did not find any other exposure factors besides tobacco smoking and inflammatory bowel disease to be causally associated with the parameters of ORA. Although the CH and CRF of KC are lower than those of healthy corneas, it has been observed that there is considerable overlap in the distribution of the two parameters, and therefore, the sensitivity and specificity of the KC diagnosis are relatively weak and do not accurately predict the changes in corneal biomechanics [54]. Different from ORA, Corvis ST analyses corneal deformation parameters based on the dynamic examination of the corneal response. It uses an ultra-high-speed Scheimpflug camera to take 140 horizontal 8 mm frames over a period of 33 ms. In particular, Corvis ST provides a more reliable representation of parameters that characterise corneal biomechanics, for example, SSI, SP-A1, CBI, etc, which have important applications in the diagnosis and treatment of KC [8]. Further MR studies with biomechanical data from, e.g., Corvis ST [55] and Brillouin optical microscopy [56] may be desirable as these technologies were shown to provide more accurate biomechanical indices to reflect the biomechanical abnormalities in KC [8] and, therefore, may promising for better explaining the bridging roles of corneal biomechanics between KC development and its exposure factors.

In conclusion, this MR study suggests that in European populations, asthma, Down syndrome, inflammatory bowel disease, atopic dermatitis, and serum 25-hydroxyvitamin D levels are associated with a higher risk of KC, while smoking is associated with a lower risk. These findings highlight the multifactorial nature of KC involving both environmental and genetic factors and emphasise the importance of timely screening in specific populations for effective KC prevention. Further MR analysis is warranted to include more representative biomechanical measures with more SNPs and KC cases.

## Figures and Tables

**Table 1:** MR results of tobacco smoking on CH and CRF.

Outcome	MR Methods	No. of SNPs	OR (95%CI)	*p*-Value	*P*-Heterogeneity	*P*-Pleiotropy
CH		20			0.360	0.192
	IVW		1.572 (1.216–2.033)	<0.001 *		
	Weighted median		1.345 (0.955–1.893)	0.090		
	MR-Egger		0.874 (0.360–2.121)	0.769		
CRF		20			0.525	0.624
	IVW		1.380 (1.086–1.755)	0.009 *		
	Weighted median		1.251 (0.881–1.777)	0.211		
	MR-Egger		1.122 (0.481–2.616)	0.792		

IVW = inverse variance weighted; MR-Egger = Mendelian randomisation–Egger; CI = confidence interval; OR = odds ratio; MR = Mendelian randomisation; SNP = single-nucleotide polymorphism; CH= corneal hysteresis; CRF= corneal resistance factor; * represents the correlation is significant at 0.05 level.

**Table 2:** MR results of inflammatory bowel disease on CH and CRF.

Outcome	MR Methods	No. of SNPs	OR (95%CI)	*p*-Value	*P*-Heterogeneity	*P*-Pleiotropy
CH (Outlier removed)		110			<0.001 *	0.722
	IVW		0.989 (0.978–0.999)	0.032 *		
	Weighted median		0.994 (0.980–1.009)	0.446		
	MR-Egger		0.984 (0.960–1.010)	0.226		
CRF (Outlier removed)		108			<0.001 *	0.879
	IVW		0.982 (0.971–0.993)	0.001 *		
	Weighted median		0.983 (0.971–0.996)	0.010 *		
	MR-Egger		0.980 (0.955–1.006)	0.137		

IVW = inverse variance weighted; MR-Egger = Mendelian randomisation–Egger; CI = confidence interval; OR = odds ratio; MR = Mendelian randomisation; SNP = single-nucleotide polymorphism; CH = corneal hysteresis; CRF = corneal resistance factor; * represents the correlation is significant at 0.05 level.

**Table 3:** MR results of smoking, asthma, down syndrome, inflammatory bowel disease, atopic dermatitis and serum 25-hydroxyvitamin D levels on the risk of KC.

Exposures	MR Methods	No. of SNPs	OR (95%CI)	*p*-Value	*P*-Heterogeneity	*P*-Pleiotropy
Current tobacco smoking	Wald ratio	1	0.055 (0.004–0.677)	0.024 *	NA	NA
Down syndrome	Wald ratio	1	3.276 (1.453–7.388)	0.004 *	NA	NA
Asthma		98			0.683	0.255
	IVW		39.901 (2.522–631.169)	0.009 *		
	Weighted median		3.273 (0.043–251.786)	0.593		
	MR-Egger		1.020 (0.001–966.600)	0.996		
Inflammatory bowel disease		111			0.303	0.723
	IVW		1.206 (1.034–1.407)	0.017 *		
	Weighted median		1.163 (0.921–1.469)	0.203		
	MR-Egger		1.132 (0.770–1.663)	0.531		
Atopic dermatitis		20			0.606	0.504
	IVW		1.452 (1.085–1.944)	0.012 *		
	Weighted median		1.302 (0.864–1.961)	0.207		
	MR-Egger		1.142 (0.539–2.417)	0.733		
Serum 25-Hydroxyvitamin D levels		104			0.327	0.930
	IVW		2.146 (1.040–4.429)	0.039 *		
	Weighted median		2.365 (0.770–7.264)	0.142		
	MR-Egger		2.218 (0.673–7.317)	0.195		

IVW = inverse variance weighted; MR-Egger = Mendelian randomisation–Egger; CI = confidence interval; OR = odds ratio; MR = Mendelian randomisation; SNP = single-nucleotide polymorphism; * represents the correlation is significant at 0.05 level; NA = not applicable.

## Data Availability

Publicly available datasets were analysed in this study. This data can be found here: [https://gwas.mrcieu.ac.uk/] (accessed on 22 February 2024).

## Supplementary Materials

The following supporting information can be downloaded at: https://www.mdpi.com/article/10.3390/bioengineering11030221/s1, Figure S1: Scatter and leave-one-out plots of tobacco smoking with Corneal Hysteresis (left eye); Figure S2: Scatter and leave-one-out plots of tobacco smoking with Corneal Resistance Factor (left eye); Figure S3: Scatter and leave-one-out plots of inflammatory bowel disease with Corneal Hysteresis (left eye); Figure S4: Scatter and leave-one-out plots of inflammatory bowel disease with Corneal Resistance Factor (left eye); Figure S5: Scatter and leave-one-out plots of asthma with keratoconus. a: Scatter plot demonstrating the effect of each asthma-associated genetic variant on keratoconus on the log-odds scale; Figure S6: Supplementary Figure S6 Scatter and leave-one-out plots of atopic dermatitis with KC; Figure S7: Scatter and leave-one-out plots of inflammatory bowel disease with KC; Figure S8: Supplementary Figure S8 Scatter and leave-one-out plots of serum 25−hydroxyvitamin D levels with KC.

## Abbreviations

KC	Keratoconus
MR	Mendelian randomisation
IVW	Inverse variance weighted
SNP	Single-nucleotide polymorphism
ORA	Ocular Response Analyzer
CVS	Corvis ST
CH	Corneal hysteresis
CRF	Corneal resistance factor
RCT	Randomised control trial
GWAS	Genome-wide association study
SSI	Stress–stain index
CBI	Sorneal biomechanical index
CEU	Cardiovascular Epidemiology Unit
IV	Instrumental variables
LD	Linkage disequilibrium
MR Egger	Mendelian randomisation–Egger
CI	Confidence interval
OR	Odds ratio
ECM	Extracellular matrix
MMPs	Matrix metalloproteinases
IL-6	Interleukin-6
TNF-α	Tumor necrosis factor-α
SP-A1	Corneal stiffness parameter at first applanation time
