# Supplementary material for: Mendelian Randomisation Analysis of Causal Association between Lifestyle, Health Factors, and Keratoconus
Source: Bioengineering (Basel). 2024 Feb 26;11(3):221. doi: 10.3390/bioengineering11030221 (PMC10968464; doi:10.3390/bioengineering11030221)
Supplement: Supplementary file 1 [file bioengineering-11-00221-s001.zip › bioengineering-2867178-supplementary.pdf]

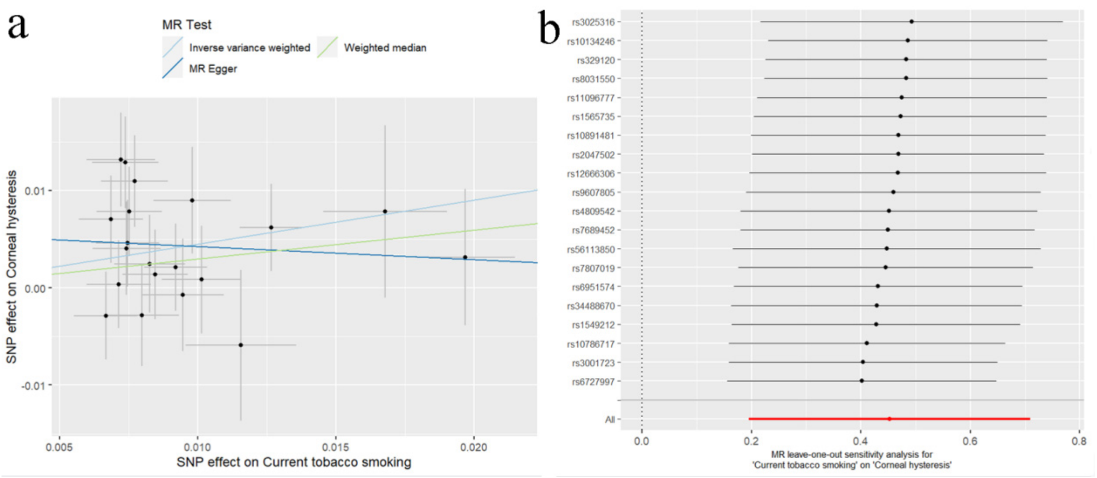


**Supplementary Figure 1** Scatter and leave-one-out plots of tobacco smoking with Corneal Hysteresis (left eye). a: Scatter plot demonstrating the effect of each tobacco smoking-associated genetic variant on Corneal Hysteresis (left eye) on the log-odds scale. The slopes of each line represent the causal association for each method. b: Leave-one-out analysis of the causal effect of tobacco smoking on Corneal Hysteresis (left eye) susceptibility. It represents the IVW MR approach for the causal effect of tobacco smoking on Corneal Hysteresis(left) susceptibility after excluding the corresponding SNPs one by one.

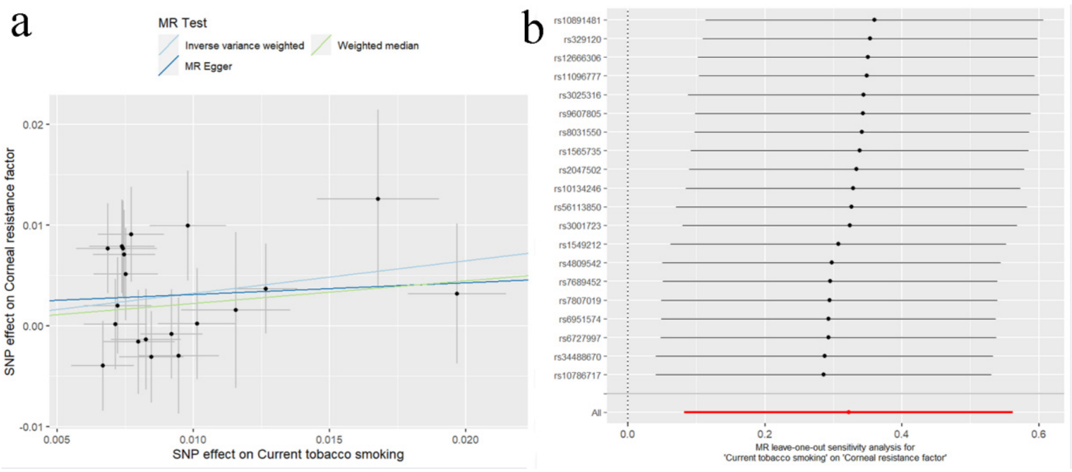


**Supplementary Figure 2** Scatter and leave-one-out plots of tobacco smoking with Corneal Resistance Factor (left eye). a: Scatter plot demonstrating the effect of each tobacco smoking-associated genetic variant on Corneal Resistance Factor(left) on the log-odds scale. The slopes of each line represent the causal association for each method. b: Leave-one-out analysis of the causal effect of tobacco smoking on Corneal Resistance Factor (left eye) susceptibility. It represents the IVW MR approach for the causal effect of tobacco smoking on Corneal Resistance Factor (left eye) susceptibility after excluding the corresponding SNPs one by one.

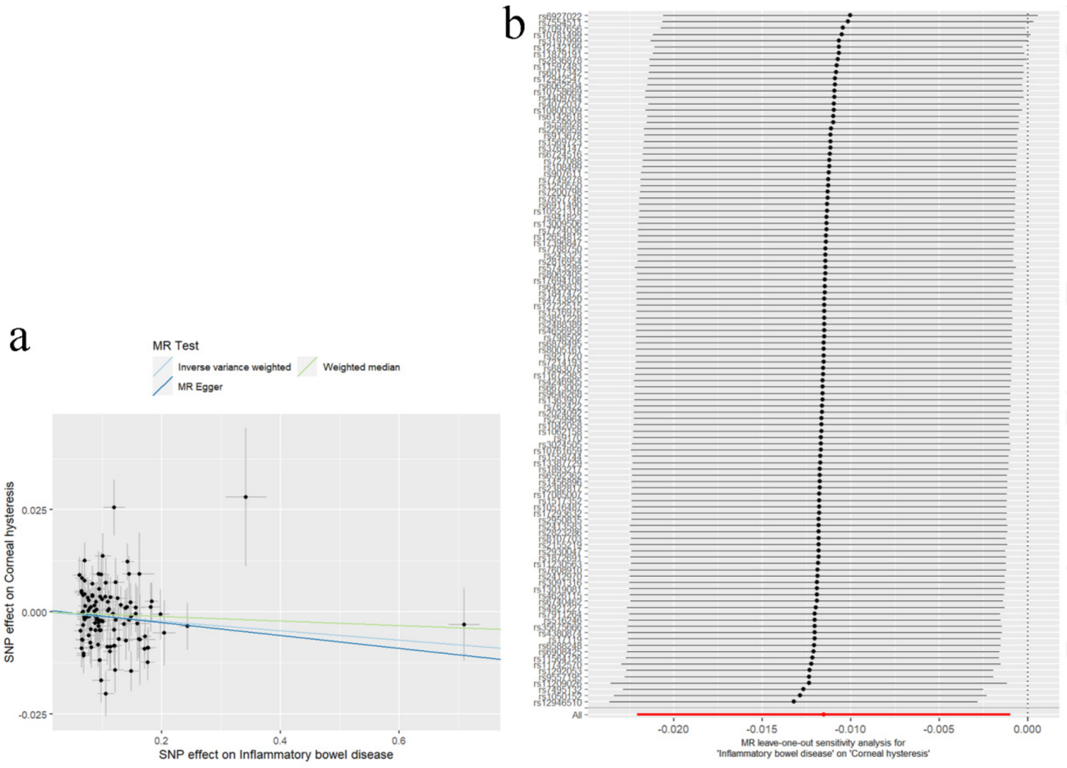


**Supplementary Figure 3** Scatter and leave-one-out plots of inflammatory bowel disease with Corneal Hysteresis (left eye). a: Scatter plot demonstrating the effect of each inflammatory bowel disease-associated genetic variant on Corneal Hysteresis (left eye) on the log-odds scale. The slopes of each line represent the causal association for each method. b: Leave-one-out analysis of the causal effect of inflammatory bowel disease on Corneal Hysteresis (left eye) susceptibility. It represents the IVW MR approach for the causal effect of inflammatory bowel disease on Corneal Hysteresis (left eye) susceptibility after excluding the corresponding SNPs one by one.

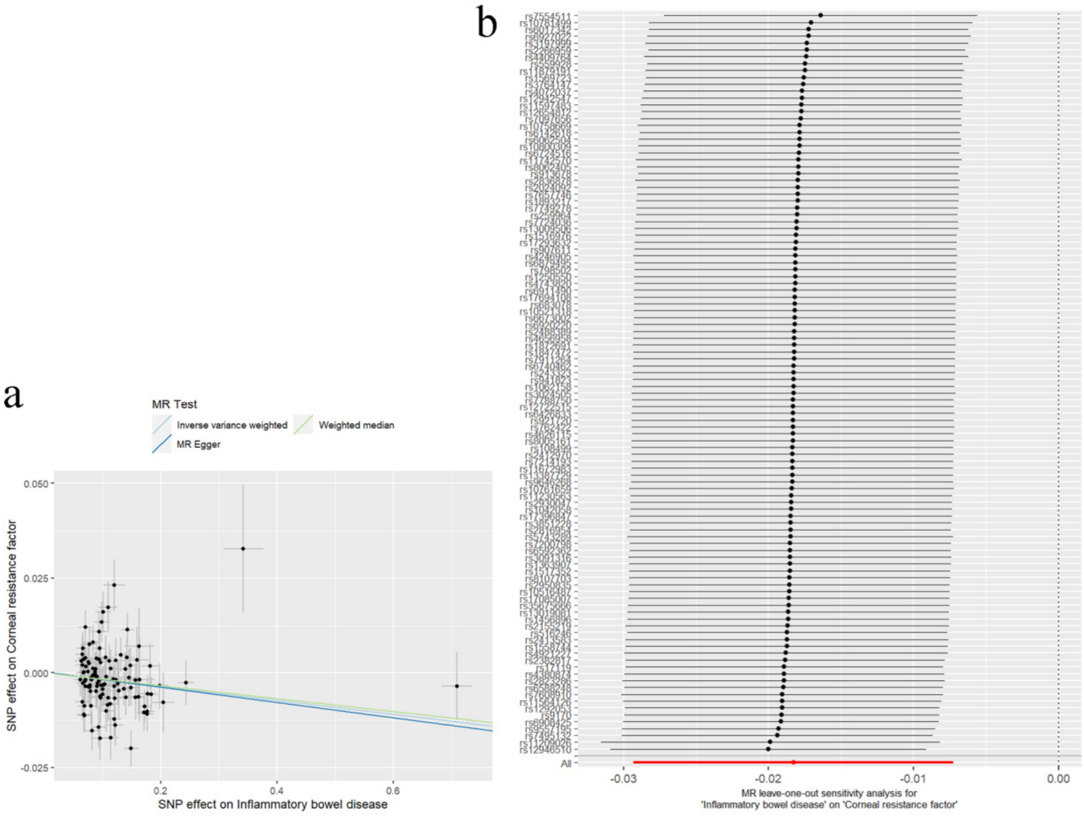


**Supplementary Figure 4** Scatter and leave-one-out plots of inflammatory bowel disease with Corneal Resistance Factor (left eye). a: Scatter plot demonstrating the effect of each inflammatory bowel disease -associated genetic variant on Corneal Resistance Factor (left eye) on the log-odds scale. The slopes of each line represent the causal association for each method. b: Leave-one-out analysis of the causal effect of inflammatory bowel disease on Corneal Resistance Factor (left eye) susceptibility. It represents the IVW MR approach for the causal effect of inflammatory bowel disease on Corneal Resistance Factor (left eye) susceptibility after excluding the corresponding SNPs one by one.

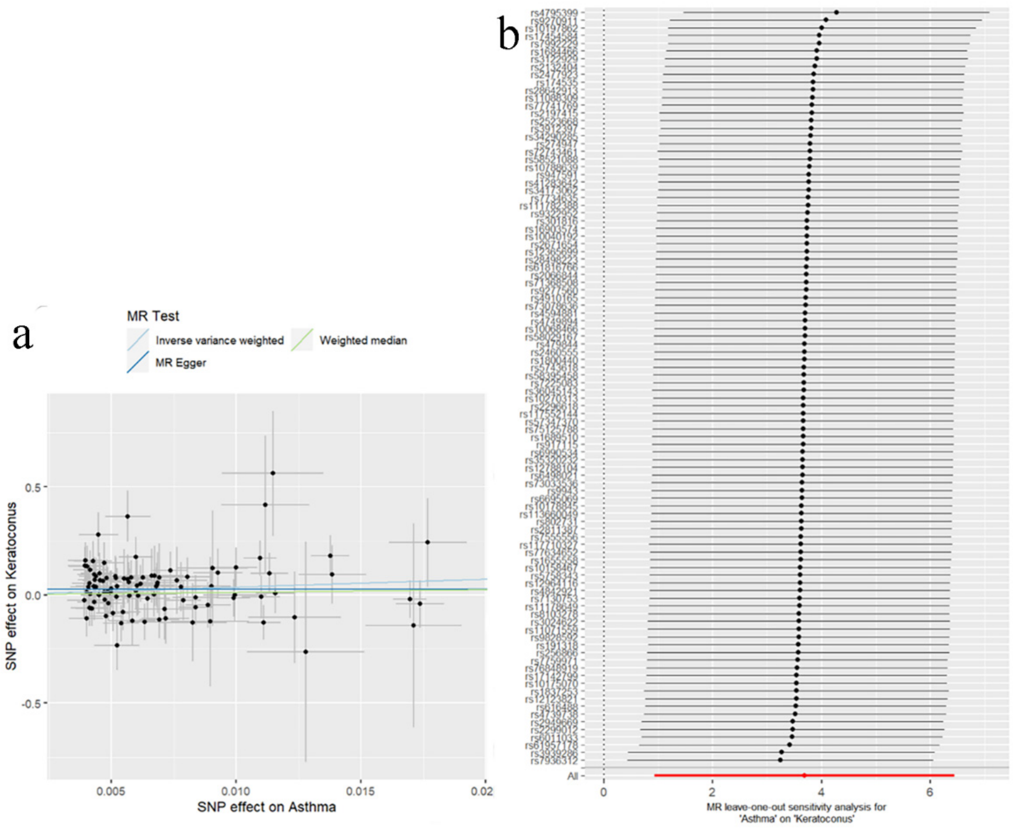


**Supplementary Figure 5** Scatter and leave-one-out plots of asthma with keratoconus. a: Scatter plot demonstrating the effect of each asthma-associated genetic variant on keratoconus on the log-odds scale. The slopes of each line represent the causal association for each method. b: Leave-one-out analysis of the causal effect of asthma on keratoconus susceptibility. It represents the IVW MR approach for the causal effect of asthma on keratoconus susceptibility after excluding the corresponding SNPs one by one.

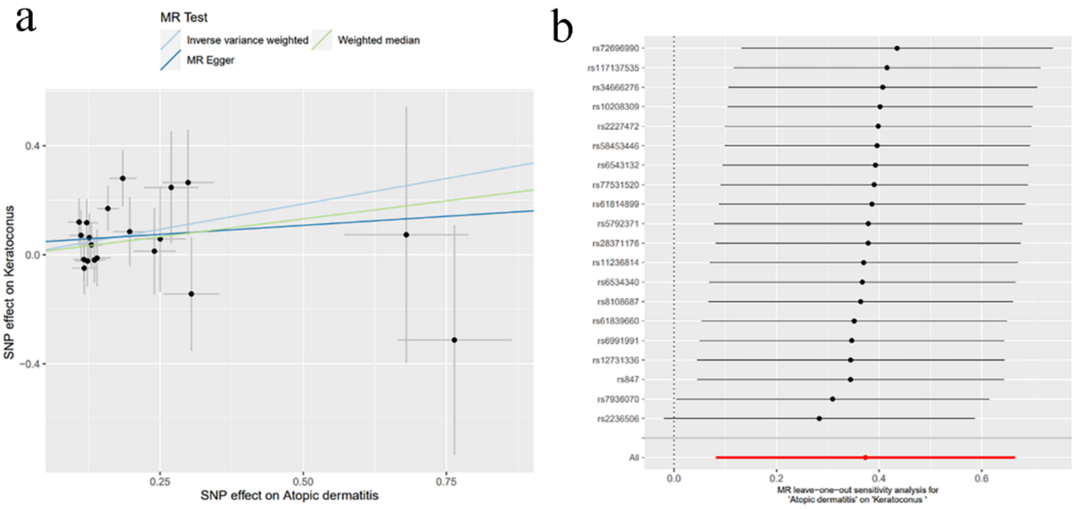


**Supplementary Figure 6** Scatter and leave-one-out plots of atopic dermatitis with KC. a: Scatter plot demonstrating the effect of each atopic dermatitis-associated genetic variant on KC on the log-odds scale. The slopes of each line represent the causal association for each method. b: Leave-one-out analysis of the causal effect of atopic dermatitis on KC susceptibility. It represents the IVW MR approach for the causal effect of atopic dermatitis on KC susceptibility after excluding the corresponding SNPs one by one.

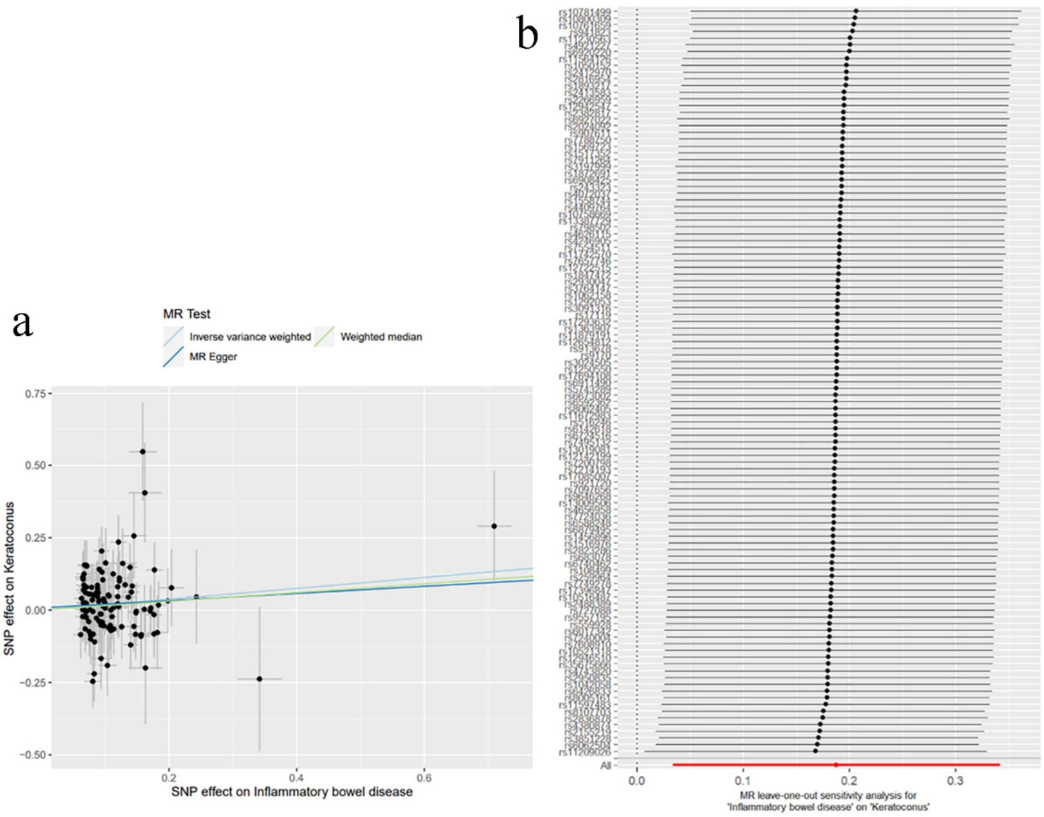


**Supplementary Figure 7** Scatter and leave-one-out plots of inflammatory bowel disease with KC. a: Scatter plot demonstrating the effect of each inflammatory bowel disease-associated genetic variant on KC on the log-odds scale. The slopes of each line represent the causal association for each method. b: Leave-one-out analysis of the causal effect of inflammatory bowel disease on KC susceptibility. It represents the IVW MR approach for the causal effect of inflammatory bowel disease on KC susceptibility after excluding the corresponding SNPs one by one.

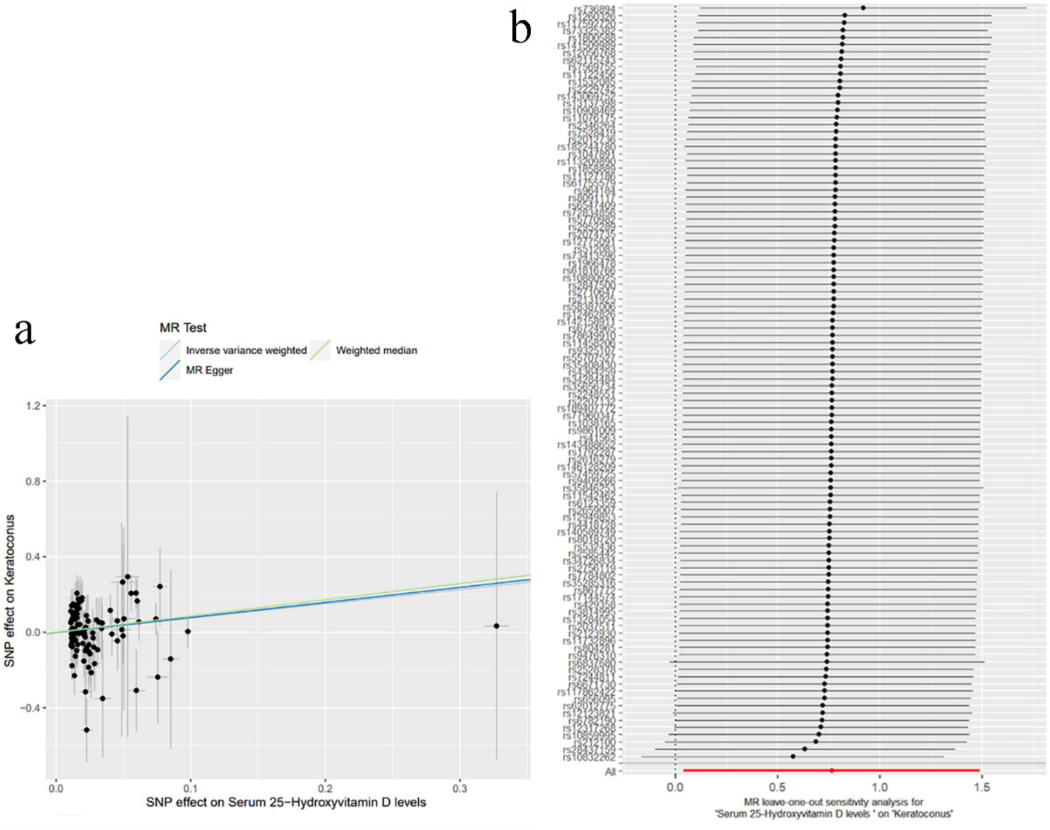


**Supplementary Figure 8** Scatter and leave-one-out plots of serum 25−hydroxyvitamin D levels with KC. a: Scatter plot demonstrating the effect of each Serum 25−Hydroxyvitamin D levels -associated genetic variant on KC on the log-odds scale. The slopes of each line represent the causal association for each method. b: Leave-one-out analysis of the causal effect of serum 25−hydroxyvitamin D levels on KC susceptibility. It represents the IVW MR approach for the causal effect of serum 25−hydroxyvitamin D levels on KC susceptibility after excluding the corresponding SNPs one by one.
